# Chemokine (C-C Motif) Ligand 20, a Potential Biomarker for Graves' Disease, Is Regulated by Osteopontin

**DOI:** 10.1371/journal.pone.0064277

**Published:** 2013-05-22

**Authors:** Xiaoli Li, Yicheng Qi, Xinran Ma, Fengjiao Huang, Hua Guo, Xiaohua Jiang, Jie Hong, Dongping Lin, Bin Cui, Guang Ning, Lingyan Xu, Shu Wang

**Affiliations:** 1 Shanghai Clinical Center for Endocrine and Metabolic Diseases, Department of Endocrinology and Metabolism, Ruijin Hospital, Affiliated to Shanghai Jiao-Tong University School of Medicine, Shanghai, China; 2 Laboratory of Endocrinology and Metabolism, Institute of Health Sciences, Shanghai Institutes for Biological Sciences (SIBS), Chinese Academy of Sciences (CAS) & Shanghai Jiao Tong University School of Medicine (SJTUSM), Shanghai, China; 3 Genetics of Development and Disease Branch, National Institute of Diabetes and Digestive and Kidney Diseases, National Institutes of Health, Bethesda, Maryland, United States of America; 4 Department of Endocrinology and Metabolism, the Ninth People's Hospital, Shanghai Jiao Tong University School of Medicine, Shanghai, China; University of Leuven, Rega Institute, Belgium

## Abstract

**Context:**

Graves’ disease (GD) is a common autoimmune disease involving the thyroid gland. The altered balance of pro- and anti-inflammatory cytokines plays an important role in the pathogenesis of GD. Chemokine (C-C motif) ligand 20 (CCL20) is important for interleukin-17 (IL-17) signal activation and a potent chemoattractant for Th17 cells. Meanwhile, Osteopontin (OPN), a broadly expressed pleiotropic cytokine, has been implicated in GD through inducing Th1-involved response to enhance the production of proinflammatory cytokines and chemokines, but little is known about the role of OPN in regulating CCL20 and IL-17 signaling.

**Objective:**

This study sought to explore the possibility of CCL20 level as a biomarker for GD, as well as investigate the role of OPN in regulating CCL20 production.

**Methods:**

Fifty untreated GD patients, fifteen euthyroid GD patients, twelve TRAb-negative GD patients and thirty-five healthy control donors were recruited. OPN, CCL20 and other clinical GD diagnosis parameters were measured. CD4+T cells were isolated from peripheral blood mononuclear cells (PBMCs) using antibody-coated magnetic beads. Enzyme-linked immune-sorbent assay and quantitative polymerase chain reaction were used to determine CCL20 expression level.

**Results:**

We found that the plasma CCL20 level was enhanced in GD patients and decreased in euthyroid and TRAb-negative GD patients. In addition, CCL20 level correlated with GD clinical diagnostic parameters and plasma OPN level. Moreover, we demonstrated that recombinant OPN and plasma from untreated GD patients increased the expression of CCL20 in CD4+T cells, which could be blocked by OPN antibody. Furthermore, we found that the effect of OPN on CCL20 expression was mediated by β3 integrin receptor, IL-17, NF-κB and MAPK pathways.

**Conclusions:**

These results demonstrated that CCL20 might serve as a biomarker for GD and suggested the possible role of OPN in induction of CCL20 expression.

## Introduction

Graves’ disease (GD) is a common organ-specific autoimmune disease characterized by the reactivity to self-thyroid antigens. Although the pathogenesis of the disease remains elusive, evidences indicated that destruction of the balance of Th1/Th2 cells and Treg/Th17 cells could alter the expressions of pro- and anti-inflammatory cytokines resulting in thyroid lymphocytic infiltration and B cell activation, with antibody production against thyroid antigens, which in turn played a pivotal role in the pathogenesis of GD [1], [2].

Th17 cell lineage, a recently described subset of CD4+T helper cells, plays a central role in initiation and pathogenesis in many autoimmune diseases [3]–[7]. The previous study demonstrated that the proportion of the Th17 cells increased in intractable GD patients, who remained positive for anti-thyrotropin receptor antibody (TRAb) despite being treated with anti-thyroid drugs [8]. Our laboratory showed the involvement of interleukin-17 (IL-17) in the etiology of GD by providing strong evidence of positive association between IL-17F polymorphisms and GD susceptibility [9]. CCL20 is first identified in the liver and can be expressed by macrophages and leukocytes [10]. It is the only chemokine known to interact with CC chemokine receptor 6 (CCR6) and responsible for chemoattractant of CCR6-positive Th17 cells [11], [12]. On the other hand, IL-17 produced from Th17 cells is also a strong inducer of CCL20 expression in many cell types [12], [13]. Thus, the positive regulatory loop indicates that CCL20 level is closely related to IL17 signal activation. Although CCL20 has been implicated in several autoimmune diseases, such as rheumatoid arthritis (RA) and Experimental Autoimmune Encephalomyelitis (EAE) [11], [12], [14], little is known about the association of CCL20 with GD and its regulatory factors.

Latest studies suggested that osteopontin (OPN) induced Th17 responses through amplification of IL-17 production, which mediated adverse effects in multiple sclerosis (MS) and RA [15], [16]. OPN, an important proinflammatory cytokine with pleiotropic functions, has been tightly linked to many autoimmune diseases, such as MS, RA and systemic lupus erythematosus (SLE) [17]–[23]. Besides, our previous study indicated that OPN was excessively produced in GD patients and acted through the NF-κB pathway to enhance the production of proinflammatory cytokines and chemokines [24]. OPN is classified as a Th1 cytokine because of its ability to enhance the production of IFN-γ from T cells and IL-12 production from macrophages [23], [25], [26]. Besides, OPN induces Th2-involved humoral immunity through up-regulation of CD40L expression, which provides a possible explanation for the ability of OPN to modulate polyclonal B cell proliferation and stimulate the production of antibodies [27]–[29]. Considering its broad function, we examined whether OPN was involved in CCL20 and IL-17 signal in GD.

In our study, we reported that plasma CCL20 level was significantly increased in GD and its expression correlated with GD clinical parameters and plasma OPN level. Moreover, we demonstrated that OPN treatment increased CCL20 expression in CD4+T cells, which might be mediated through IL-17, as well as the NF-κB and MAPK pathways.

## Materials and Methods

### Ethics Statement

This study was approved by the Institutional Review Board of the Ruijin Hospital, Shanghai Jiao Tong University School of Medicine. The written informed consent was obtained from each participant.

### Subjects

The patients in this study were recruited from the outpatient Department of Ruijin Hospital affiliated to Shang-hai Jiao Tong University. Fifty untreated GD patients (uGD), 15 euthyroid GD patients (eGD), 12 TRAb-negative GD patients (nGD) and 35 age and gender matched healthy control donors (hCD) were selected.

The criteria for selection of untreated Graves’ disease patients includes the following: patients are naïve to any treatment; the presence of typical symptoms, such as heat intolerance, fatigue, increased appetite, increased sweating, weight loss, muscle weakness, and tremors; thyroid gland was diffusely enlarged; laboratory diagnosis including decreased serum sensitive TSH (sTSH), increased free triiodothyronine (FT3), thyroxine (FT4) and TSH receptor antibody (TRAb). eGD patients were uGD patients treated with methimazole (MMI) for 1–3 months until reaching normal TSH, FT3, and FT4 values as the euthyroid state but TRAb level was just slightly decreased compared to initial GD patients. nGD patients were uGD patients treated with MMI for 1–2 years until TSH, FT3, FT4 as well as TRAb decreased to normal range and maintained stable for at least three months. hCDs were enrolled based on history, normal physical examination, measurements of thyroid hormones, and thyroid autoantibodies, thyroid ultrasonography and excluded the presence of thyroid disorders.

Thyroid gland examination by neck ultrasonography was performed by the same operator in department of ultrasound, Ruijin Hospital, who was unaware of the results of GD diagnosis. FT3, FT4, TSH and thyroperoxidase antibody (TPOAb) were measured by automated chemiluminescent immunoassays (Architect i2000SR; Abbott Laboratories). Thyroglobulin antibody (TGAb) and TRAb were measured by radioreceptor assays with commercial kits (DiaSorin, Stillwater). Demographic and clinical information of the study subjects is presented in Table 1.

**Table 1:** Clinical characteristics of the subjects.

	hCD	uGD	eGD	nGD
N	35	50	15	12
Female, n (%)	28(80%)	38(76%)	12(80%)	9(75%)
Age (y)	34.72±16.87	36.04±12.45	37.56±13.34	33.68±15.3
FT3(pmol/l)	4.61±0.52	25.18±8.81^a^	3.87±1.15b1	4.32±0.86^c^
FT4(pmol/l)	14.30±1.82	42.01±12.69^a^	12.67±3.45b1	13.56±2.05^c^
s-TSH(mIU/l)	1.58±0.81	0.0073±0.0045^a^	2.10±1.56b1	1.48±0.67^c^
TRAb(IU/L)	0.31±0.06	24.94±10.72^a^	19.62±8.73b2	0.46±0.38^c^
TPOAb(IU/ml)	0.41±0.53	191.90±224.67^a^	102.19±156.50b2	32.45±12.37^c^
TGAb(IU/ml)	2.05±4.62	115.01±165.41^a^	97.97±78.90b2	30.75±45.07^c^

a *P*<0.001, untreated GD (uGD) compared with healthy Control Donors (hCD).

b1 *P*<0.001,

b2 *P*>0.05, euthyroid GD (eGD) compared with uGD.

c *P*<0.001,TRAb-negative GD patients (nGD) compared with uGD. All data were presented as mean±SD.

### Isolation, Culture and Stimulation of PBMCs

The fresh PBMCs from a total of 77 patients including 50 uGD, 15 eGD, 12 nGD patients and 35 healthy individuals were all isolated by a Ficoll density gradient centrifugation (Sigma-Aldrich) and the viability was over 98% determined by trypan blue staining. Then, CD4+T cells were immediately purified by positive selection for further gene expression analysis. Besides, PBMCs obtained from randomly selected 8 out of 50 uGD patients and 8 out of 35 healthy individuals were prepared for cell culture. PBMCs were cultured in 6-well plates at 3×10^6^ cells per well in RPMI 1640 medium containing 10% fetal bovine serum and stimulated with plasma from uGD patients or hCDs at a dilution of 1∶4. To determine the effect of OPN, PBMCs were stimulated with human recombinant OPN (rOPN, 1433-OP-050), the time course and the dose-dependent patterns of CCL20 and IL-17 expression were analyzed. For blocking experiments, purified antibodies against human OPN (Human OPN Affinity Purified Polyclonal Ab, AF1433, 5µg/ml), human β3 receptor (Human Integrin alpha V beta 3 MAb, MAB3050, 5µg/ml), human IL-17 (Human IL-17 Affinity Purified Polyclonal Ab, AF-317-NA, 1µg/ml) and isotype-matched control antibodies (all purchased from R&D Systems) were added. For experiments involving antagonist treatment, PBMCs were pre-incubated with inhibitors (all purchased from Calbiochem) of the IκB kinase (IKK2 inhibitor, SC-514), Mitogen-activated protein kinase (MAPK) including p38 (MAP Kinase Inhibitor, 506126), JNK (JNK Inhibitor II, 420119), MEK-1/2 (MEK-1/2 inhibitor PD98059) and PI3K (Wortmannin, PI3K inhibitor II, 681675) for 30 minutes, followed by stimulation with rOPN (1 µg/ml) for 12 hours. After treatment, the viability of PBMCs was around 95% determined by trypan blue staining. CD4+T cells were then purified from these cultured PBMCs for gene expression analysis.

### CD4+T cells Purification

For purification of CD4+T cell from fresh or cultured PBMCs for gene expression analysis, positive selection by human CD4 Micro Beads (Miltenyi Biotec) was used according to the manufacturer’s instructions. The purity of CD4+T cells was over 95% examined by FACSAria cytometer (BD Biosciences).

### RNA Extraction and Quantitative RT-PCR

All CD4+T cell specimens were snap frozen upon extraction and stored at −80 degrees until use. Total RNA was isolated from CD4+T cell samples using TRIZOL reagent (Invitrogen) and 1 mg total RNA was converted into first-strand cDNA with the First Strand cDNA Synthesis Kit (Promega) according to the manufacturer’s instructions. Quantitative RT-PCR was performed using SYBR Master Mix (Takara) on an ABI Prism 7900 HT (Applied Biosystems). BLAST searches were conducted on the primer nucleotide sequences to ensure gene specificity. PCR primers were as follows: for human GAPDH, forward 5′- TGTTGCCATCAATGACCCCTT-3′ and reverse 5′CTCCACGACGTACTCAGCG-3′; for human CCL20 forward 5′-TGCTGTACCAAGAGTTTGCTC-3′ and reverse 5′- CGCACACAGACAACTTTTTCTTT-3′; for human IL-17 forward 5′- TCCCACGAAATCCAGGATGC-3′ and reverse 5′- TGTTCAGGTTGACCATCACAGT-3′. The PCR measurements were performed in triplicates and the results were calculated by the ΔΔCt method and normalized against endogenous control gene GAPDH.

### ELISA

Plasma from all 77 patients, including 50 uGD, 15 eGD and 12 nGD patients and 35 hCDs were used for ELISA analysis. Peripheral blood from each individual was collected in a sodium-heparin vacutainer tube, and then centrifuged at 3,000 rpm for 5 minutes. Plasma from supernatant was collected and immediately stored at −80°C until used. The culture medium for treated PBMCs was also stored at −80°C before used. The CCL20, OPN and IL17 levels in plasma or cell culture medium were measured in duplicates by various ELISA kits (R&D Systems) according to the manufacturer’s instructions. The minimum detectable dose of each kit is 0.006 ng/ml for OPN, 0.10–0.87 pg/ml for CCL20 and 15 pg/ml for IL-17.

### Statistical Analysis

Statistical analysis was performed using SAS version 8.1(SAS Institute, Cary, NC). Normally distributed variables were presented as mean±SD, otherwise median and interquartile ranges were presented. Differences among normally distributed groups were tested by one-way ANOVA, and post hoc comparisons were performed with Dunnett-Bonferroni test. The plasma OPN and CCL20 levels were logarithmically transformed due to a non-normal distribution. Pearson’s correlations were first performed to evaluate the associations between CCL20 or OPN and classic GD diagnostic parameters. Those variables which were significant at *P*<0.20 level in Pearson’s correlations were further put into the multivariate forward-stepwise linear regression models to identify the factors that were independently associated with plasma CCL20 or OPN level. The OPN and CCL20 measurements shown as mRNA expression level in cells or protein level in cell culture medium were presented as mean±SEM in bar graphs. Student’s test was used to analyze the differences of realtime PCR results between two groups. Two-sided *P* values less than 0.05 were considered statistically significant.

## Results

### Plasma CCL20 Level and CCL20 mRNA Expression in CD4+T cells of GD Patients

**Figure 1:**
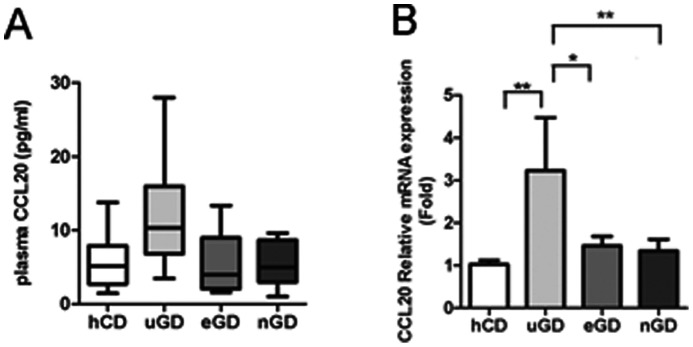
Plasma CCL20 and CCL20 mRNA in CD4+T cells from GD patients and normal controls. (A) Distribution of plasma CCL20 levels in 35 healthy control donors, 50 untreated GD patients, 15 euthyroid GD patients and 12 TRAb-negative GD patients. Median values, interquartile ranges and ranges are denoted by horizontal bars, boxes and vertical bars, respectively. (B) CCL20 mRNA levels in CD4+T cells from healthy controls, uGD, eGD and nGD patients. Data were presented as mean±SEM.*, *P*<0.05; **, *P*<0.01.

As shown in Fig. 1A, plasma CCL20 levels in uGD patients were significantly higher than those from healthy control donors. [median, 10.32 (interquartile range, 6.815–15.97) pg/ml measured in 50 uGD patients versus 5.143 (interquartile range 2.712–7.892) pg/ml measured in 35 hCD; *P*<0.05]. Compared with uGD patients, plasma CCL20 levels were reduced in eGD patients [3.940 (interquartile range 2.059–8.969) pg/ml, *P*<0.05] and nGD patients [5.006 (interquartile range 2.993–8.673) pg/ml, *P*<0.05]. Consistently, CCL20 mRNA expression in CD4+T cells increased in uGD patients compared to healthy subjects but further suppressed in GD remission patients (Fig. 1B). Moreover, Person’s correlation analysis revealed that CCL20 level correlated with the majority of clinical GD parameters including FT3, FT4, TSH, TRAb, and TGAb (Table 2). After performing multivariate stepwise linear regression analysis, we found that CCL20 level was positively correlated with FT4 and TGAb (Table 2). These results showed that plasma CCL20 level was elevated in GD patients and its level correlated with GD parameters.

**Table 2:** Pearson’s correlation and multiple stepwise linear regression analysis of CCL20 associated with classic GD diagnostic parameters.

Log CCL20	*r*	*P* value	β±SE	*P* value
FT3	0.51	<0.0001		
FT4	0.56	<0.0001	0.0088±0.0018	<0.0001
TSH	−0.45	0.0002		
TRAb	0.31	<0.014		
TGAb	0.35	0.001	0.0003±0.00013	0.031
TPOAb	0.31	0.015		

*r*, correlation coefficient; β, Regression coefficient; SE, standard error.

### Correlation of Plasma CCL20 Level with OPN Level in GD

**Figure 2:**
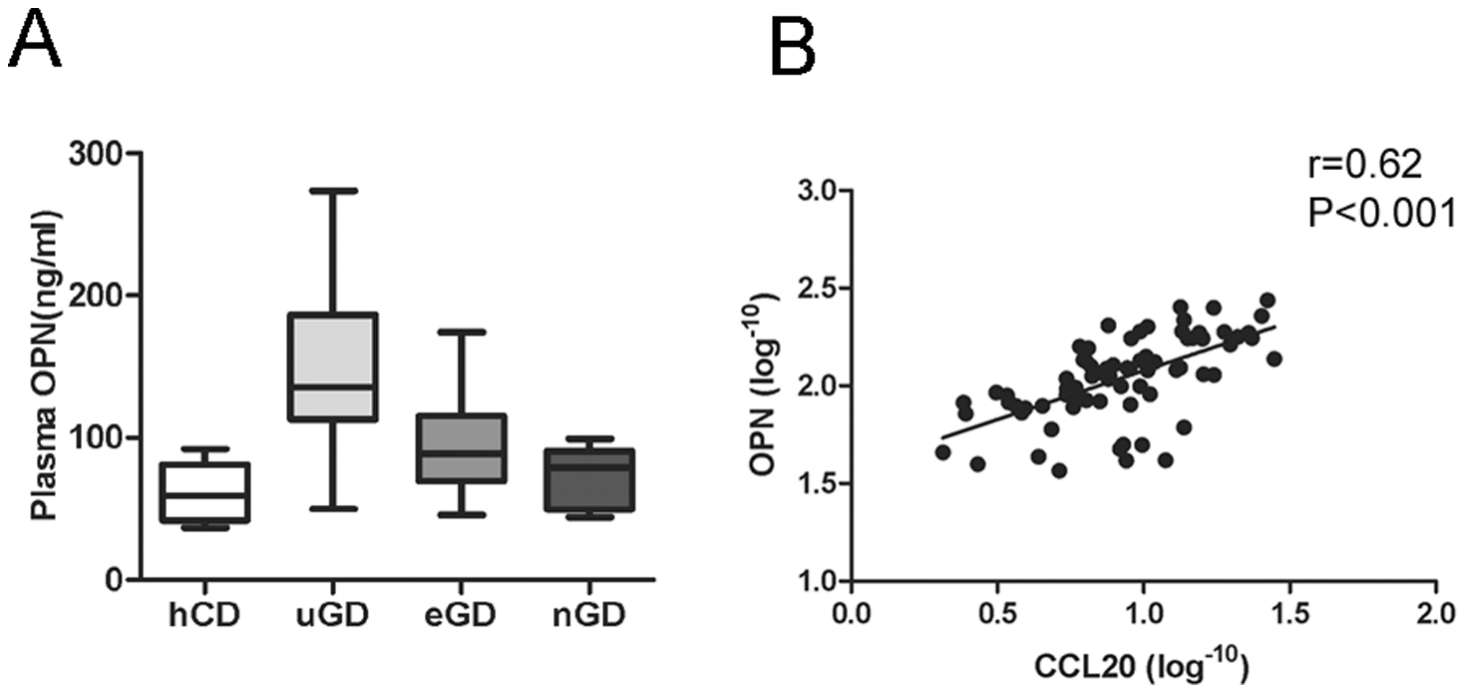
Plasma OPN levels in GD patients and normal controls and the correlation between plasma CCL20 with OPN. (A) Distribution of plasma OPN levels in 35 healthy control donors, 50 untreated GD patients, 15 euthyroid GD patients and 12 TRAb-negative GD patients. Median values, interquartile ranges and ranges are denoted by horizontal bars, boxes and vertical bars, respectively. (B) Regression scatter plots showing the correlation between logarithm-transformed plasma CCL20 level and logarithm-transformed plasma OPN level. The correlation shown was significant (*r* = 0.62, *P*<0.001).

Consistent with our previous findings (24), plasma concentrations of OPN were significantly increased in uGD patients compared with hCD [135.7 (interquartile range 112.9–186.5) ng/ml measured in uGD patients versus 59.41 (interquartile range 42.03–81.53) ng/ml measured in hCD; *P*<0.001). Moreover, we found that OPN level significantly reduced after medical treatment in eGD patients [88.86 (interquartile range 69.5–115.5) ng/ml, *P*<0.01] and nGD patients [78.91 (interquartile range 49.85–90.75) ng/ml, *P*<0.001] compared with uGD patients (Fig. 2A). Besides, plasma OPN level also correlated with clinical parameters of GD (Table 3).

**Table 3:** Pearson’s correlation and multiple stepwise linear regression analysis of OPN associated with classic GD diagnostic parameters.

Log OPN	*r*	*P* value	β±SE	*P* value
FT3	0.64	<0.0001		
FT4	0.66	<0.0001	0.0058±0.0016	0.0006
TSH	−0.52	<0.0001	−0.035±0.015	0.027
TRAb	0.49	<0.001	0.0035±0.0018	0.050
TGAb	0.27	0.0056		
TPOAb	0.34	0.0064		

*r*, correlation coefficient; β, Regression coefficient; SE, standard error.

The correlation coefficient between plasma CCL20 and OPN was then calculated. Plasma OPN and CCL20 levels were logarithmically transformed before Pearson's correlation analysis due to their non-normal distributions. Interestingly, the plasma level of CCL20 displayed a significant correlation with the plasma level of OPN (*r* = 0.62, *P*<0.001) (Fig. 2B). Besides, another multivariate stepwise linear regression analysis using CCL20 as the dependent variable and OPN and other clinical parameters as covariates showed that CCL20 significantly correlated with OPN (β = 0.80, SE = 0.15, *P*<0.01) and TGAb (β = 0.0026, SE = 0.00013, *P*<0.05). The positive correlation between plasma OPN and CCL20 concentrations suggested that OPN might be connected with CCL20.

### Induction of CCL20 by OPN in GD Plasma and Human Recombinant OPN

**Figure 3:**
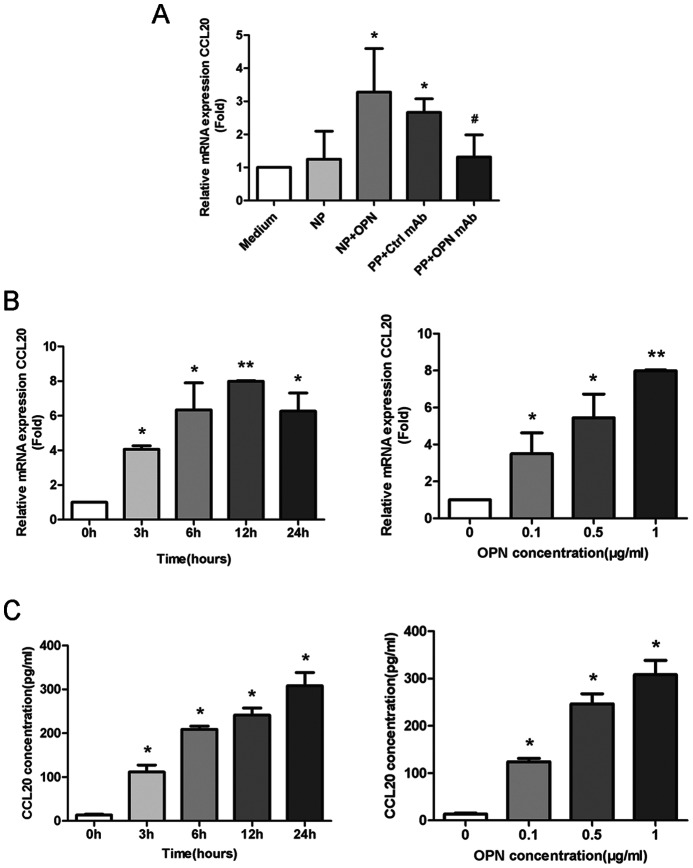
OPN induced CCL20 expressions in CD4+T cells. (A) Induction of CCL20 level in plasma from uGD patients and healthy controls. Medium: RPMI 1640; NP, normal plasma; NP+ OPN: normal plasma added with 0.1 µg/ml OPN; PP, patient plasma; PP+OPNmAb : patient plasma pretreated with 5 µg/ml OPN-neutralizing antibody for 30 min. *, *P*<0.05 versus medium control; #, *P*<0.05 versus Ctrl mAb. (B, C) OPN induced CCL20 mRNA expressions (B) and protein levels (C) in a time and dose-dependent manner. For time course, the PBMCs were cultured in the presence of human recombinant OPN (1 µg/ml) and analyzed at the indicated time points. To determine the dose dependent manner of CCL20 expression, the PBMCs were cultured for 12 hours with OPN at the indicated concentrations. CD4+T cells and culture medium were then purified and analyzed. Shown are representative results from 3 independent experiments with separate specimens. All data were presented as mean±SEM. *, *P*<0.05; **, *P*<0.01, versus medium control.

We then tested whether the elevated plasma OPN was responsible for the up-regulation of CCL20 in peripheral blood CD4+ T cells in GD patients. PBMCs from healthy donors were cultured with plasma from uGD patients or from healthy donors. Interestingly, we found increased CCL20 expression in CD4+T cells purified from PBMCs treated with plasma from uGD patients, but not in cells treated with plasma from healthy donors (Fig. 3A). Treatment with anti-OPN mAb significantly suppressed the up-regulation of CCL20 mRNA expression, indicating that OPN was involved in CCL20 up-regulation (Fig. 3A). Indeed, we demonstrated that rOPN induced CCL20 mRNA and protein levels in a time- and dose-dependent manner. (Fig. 3B, C). These results indicated that OPN might induce CCL20 expression in CD4+T cells.

### OPN Induced CCL20 Production by CD4+T cells was Mediated through β3 Integrin Receptor

**Figure 4:**
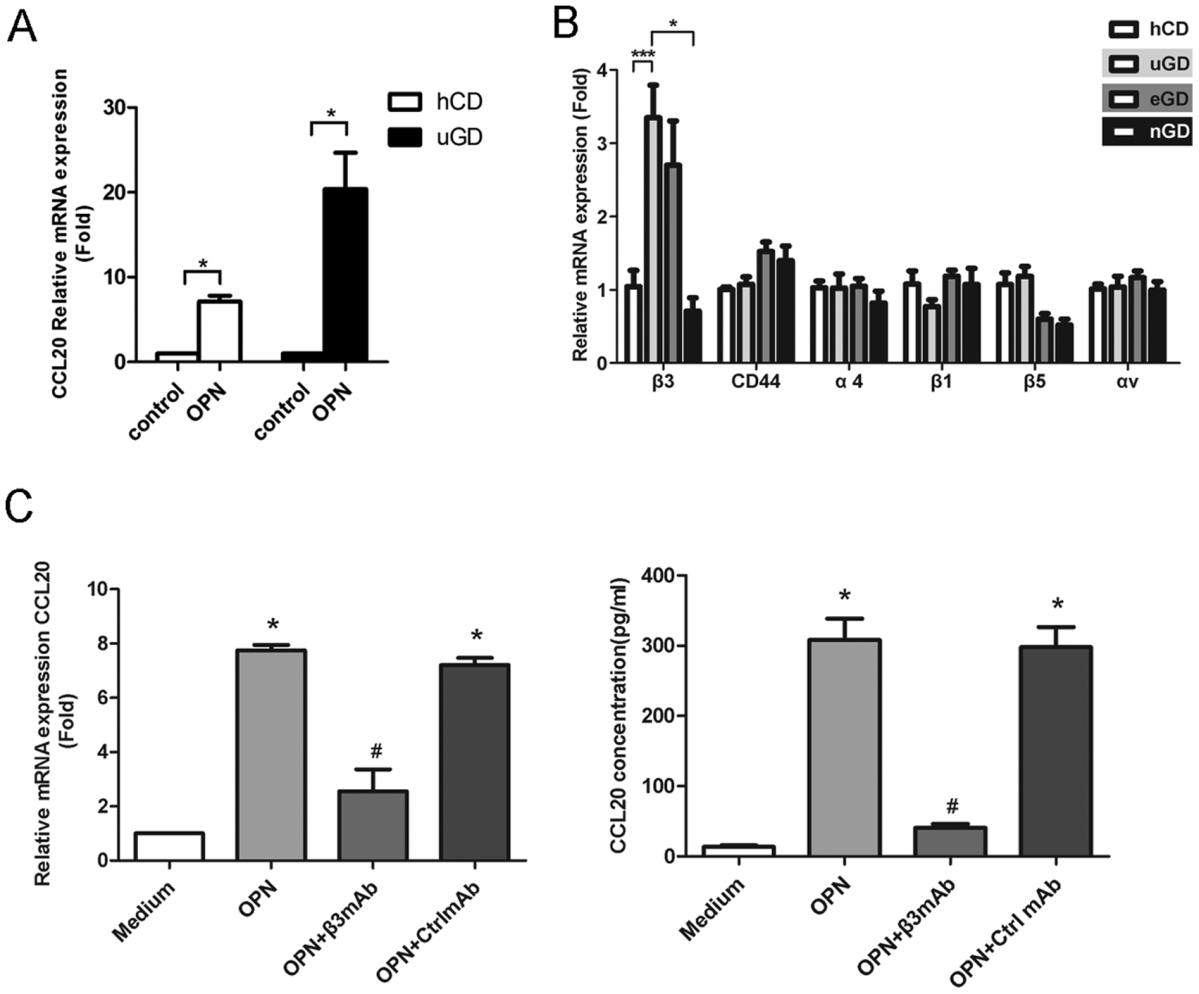
β3 integrin receptor medicated OPN induction of CCL20 expressions. (A) CCL20 gene expressions in isolated CD4+T cells from 8 hCD and 8 uGD in the presence or absence of 1 µg/ml rOPN for 12 h. (B) Expressions of OPN receptors on CD4+ T cells from uGD patients, eGD patients, nGD patients and hCD. (C) β3 integrin receptor antibody blocked induction of CCL20 mRNA and protein levels by OPN. The freshly isolated PBMCs were cultured in the presence or absence of 1 µg/ml rOPN for 12 h. Blocking Abs to integrin β3, or its isotype control mAb (IgG) were added at 5 µg/ml. Supernatants from cultures and CD4+T cells separated from PBMCs were used to analyze the expression levels of CCL20. Shown are representative results from 3 independent experiments with separate specimens. All data were presented as mean±SEM. *, *P*<0.05; ***, *P*<0.001, versus medium control; #, *P*<0.05 versus Ctrl mAb.

During our experiments, we found that the OPN treatment on CD4+T cells isolated from uGD patients induced more CCL20 expressions than those from normal subjects (Fig. 4A), which might be due to inductions of OPN receptors in uGD patients. We then examined the expressions of several known integrin subunits of OPN receptors. Indeed, we found that β3 integrin receptor was specifically highly expressed on CD4+T cells of uGD patients than normal controls (Fig. 4B). Besides, β3 antibody could block the effect of OPN in the induction of mRNA and protein expression of CCL20, suggesting that OPN-induced CCL20 production in CD4+T cells might be mediated through β3 receptor (Fig. 4C).

### Effect of OPN in the Induction of CCL20 was Mediated through Inflammatory IL-17, NF-κB and MAPK Pathways

**Figure 5:**
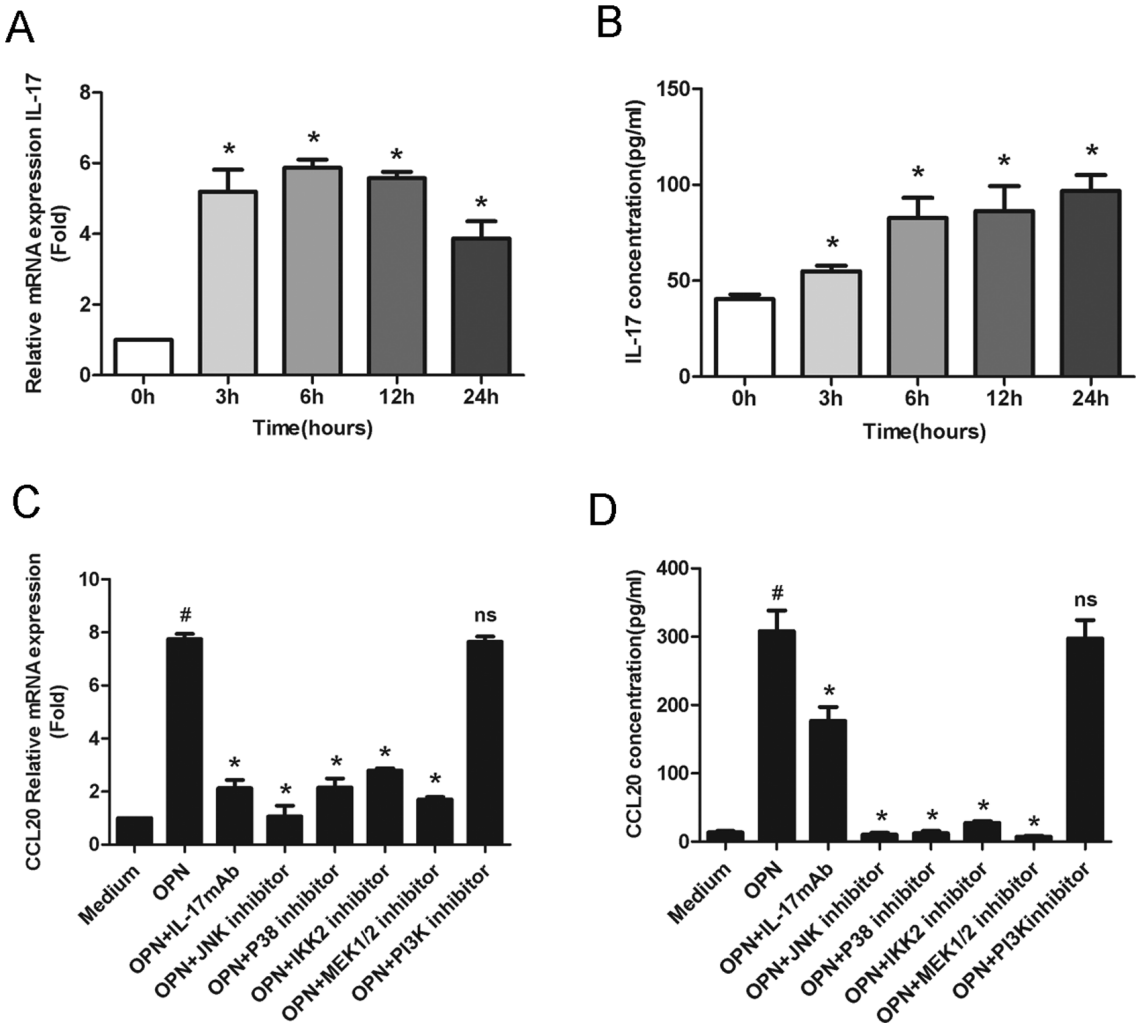
Effect of OPN on induction of CCL20 expression was mediated by IL-17, NF-κB and MAPK pathways. (A, B) PBMCs were isolated from normal subjects and treated with rOPN (1 µg/ml) at different time point. The IL-17 mRNA expression in purified CD4+T cells (A) and protein level in culture medium (B) were analyzed. Induction of CCL20 mRNA in CD4+T cells (C) and protein levels in culture medium (D) by OPN was blocked by antibody against IL-17, and inhibitors of IKK and MAPKs. Shown are representative results from 3 independent experiments with separate specimens. All data were presented as mean±SEM. *, *P*<0.05 versus OPN; # *P*<0.05 versus medium control; ns>0.05 versus OPN.

We further tried to elucidate in which possible way OPN regulated CCL20 expression. Consistent with previous reports [17], [18], we found that OPN treatment increased IL-17 production in CD4+T cells (Fig. 5A, B). Latest studies suggested that IL-17 could stimulate CCL20 production [30], [31]. Thus, we tested whether IL-17 was involved in CCL20 over-expression induced by OPN. Indeed, the induction of CCL20 by OPN was, at least partially, suppressed by anti-IL-17 monoclonal antibody (Fig. 5C, D).

Furthermore, previous studies suggested that OPN exerted its function through several signaling pathways, including IKK-IκB-NF-κB, MAPK and Akt/PI3K pathways [32]–[34]. We then examined whether these pathways participated in OPN induced CCL20 production. PBMCs derived from healthy individuals were pre-incubated with inhibitors of IKK, p38, JNK, MEK-1/2, PI3K, or with medium alone for 30 minutes, followed by stimulation with rOPN. As shown in Fig. 5C, D, the effect of OPN in the induction of CCL20 mRNA expression in CD4+T cells and protein secretion in the medium was selectively inhibited by antagonists specific for IKK and MAPK pathway including p38, JNK and MEK-1/2, but not for PI3K, suggesting the involvements of NF-κB and MAPK pathways in this regulatory process.

## Discussion

The present study provided evidences that expression of CCL20 was enhanced in uGD patients and decreased in eGD and nGD patients. In addition, CCL20 level strongly correlated with GD clinical diagnostic parameters, suggesting CCL20 might serve as a novel biomarker for GD. Besides, based on our previous study showing that OPN triggered NF-κB signaling pathway to enhance the production of proinflammatory cytokines and chemokines [24], we showed that OPN might be involved in the induction of CCL20. However due to the limitation of human study, more studies should be performed to examine the detailed molecular mechanism in vitro to fully elucidate the regulatory network.

It was reported that CCL20 interaction was required for the migration of CCR6-expressing Th17 cells to initiate self-destructive immune reactions in the joints [12]. Besides, Th17 cells regulated CCL20 expression in a positive feedback way through IL-17. Though our results showed that OPN treatment increased CCL20 and IL-17 expressions in CD4+T cells in a dose and time dependent manner (Figure 3B and C, Figure 5A and B), the study did not provide direct evidence that OPN might induce differentiation of Th17 cells in the peripheral blood and thyroid glands or attract Th17 migration to thyroid glands of GD patients. The relationship between OPN and CCL20/CCR6/Th17 axis in GD still need to be investigated. Besides, further work on evaluation of cytokines present in thyroid glands rather than circulating cytokines may provide more straightforward evidences about the association between OPN and CCL20 in GD.

CCL20 belongs to CC chemokine family. The major role of chemokines is to act as a chemoattractant to guide cell migration. Chemokines are divided into the hemeostatic or inflammatory category by their function [35]. Homeostatic chemokines, such as CCL20, are constitutively produced in certain tissues and are responsible for basal leukocyte migration. Inflammatory chemokines, for example, CCL2, CCL3 and CXCL10, are elevated by proinflammatory stimuli and help orchestrate innate and adaptive immune responses [36]. We and other groups have reported that OPN is able to induce both homeostatic and inducible chemokines expressions in PBMCs [24], [37]. Moreover, OPN is classified as Th1 cytokine because of its ability to enhance IFN-γ level, while IFN-γ inducible CXC chemokines, such as CXCL9, CXCL10 and CXCL11, have been associated with Th1-mediated immune responses and play important roles in the initial phases of autoimmune thyroid disorders [38]–[40]. Thus, it would be interesting to investigate the possible regulatory relationships between OPN and other chemokines.

It is known that OPN interacts with a variety of cell surface receptors, including αvβ3, αvβ1, α4β1, α8β1, and α9β1 integrins as well as CD44 to exert its function [41]. Binding of OPN to these cell surface receptors stimulates cell adhesion, chemotactic migration, and cytokine production [17], [42]. In this study, we found that only subunit β3 expression was specifically increased on CD4+T cells from uGD compared with other groups. Consistently, it was reported that T cells from β3 integrin deficient mice were unresponsive to OPN stimulation [15]. Besides, it was shown that IL-17 production was up-regulated by OPN through its specific interatction with β3 integrin receptor in EAE mice. [15]. Thus, the binding of OPN and β3 integrin receptor in CD4+T cells might be especially vital for its function to increase both CCl20 and IL-17 level, leading to enhanced IL-17 signaling transduction.

In summary, we found that circulating CCL20 level was elevated in GD patients and CCL20 level was associated with clinical parameters of GD and OPN level. Further study demonstrated that OPN was able to induce IL17 and CCL20 expressions in CD4+T cells. The possible effect of OPN in induction of CCL20 might go through β3 integrin receptor, IL-17, NF-kB and MAPK pathways.
